# Man, Moral and Physical

**Published:** 1860-05

**Authors:** 


MAN, MORAL AND PHYSICAL.—Such is the title of a work recently issued by the Messrs. Martien, of Chestnut Street. It is from the pen of the Rev. Dr. Joseph H. Jones, an eminent Presbyterian divine of this city, and is intended to exhibit the influence of health and disease on religious experience. It is our design to revert to it at some future period with a view to an analysis of some of its more striking contents.

